# Minimising school disruption under high incidence conditions due to the Omicron variant in France, Switzerland, Italy, in January 2022

**DOI:** 10.2807/1560-7917.ES.2023.28.5.2200192

**Published:** 2023-02-02

**Authors:** Elisabetta Colosi, Giulia Bassignana, Alain Barrat, Bruno Lina, Philippe Vanhems, Julia Bielicki, Vittoria Colizza

**Affiliations:** 1 Sorbonne Université, INSERM, Pierre Louis Institute of Epidemiology and Public Health, Paris, France; 2 Aix Marseille Univ, Université de Toulon, CNRS, CPT, Turing Center for Living Systems, Marseille, France; 3 National Reference Center for Respiratory Viruses, Department of Virology, Infective Agents Institute, Croix-Rousse Hospital, Hospices Civils de Lyon, Lyon, France; 4 Centre International de Recherche en Infectiologie (CIRI), Virpath Laboratory, INSERM U1111, CNRS—UMR 5308, École Normale Supérieure de Lyon, Université Claude Bernard Lyon, Lyon University, Lyon, France; 5 Service d'Hygiène, Épidémiologie, Infectiovigilance et Prévention, Hospices Civils de Lyon, Lyon, France; 6 Centre International de Recherche en Infectiologie (CIRI), Public Health, Epidemiology and Evolutionary Ecology of Infectious Diseases (PHE3ID) – Inserm - U1111 - UCBL Lyon 1 - CNRS –UMR5308 - ENS de Lyon, Lyon, France; 7 Paediatric Infectious Diseases, University of Basel Children's Hospital, Basel, Switzerland

**Keywords:** COVID-19, schools, testing, resources, vaccination, Omicron

## Abstract

**Background:**

As record cases of Omicron variant were registered in Europe in early 2022, schools remained a vulnerable setting undergoing large disruption.

**Aim:**

Through mathematical modelling, we compared school protocols of reactive screening, regular screening, and reactive class closure implemented in France, in Baselland (Switzerland), and in Italy, respectively, and assessed them in terms of case prevention, testing resource demand, and schooldays lost.

**Methods:**

We used a stochastic agent-based model of SARS-CoV-2 transmission in schools accounting for within- and across-class contacts from empirical contact data. We parameterised it to the Omicron BA.1 variant to reproduce the French Omicron wave in January 2022. We simulated the three protocols to assess their costs and effectiveness for varying peak incidence rates in the range experienced by European countries.

**Results:**

We estimated that at the high incidence rates registered in France during the Omicron BA.1 wave in January 2022, the reactive screening protocol applied in France required higher test resources compared with the weekly screening applied in Baselland (0.50 vs 0.45 tests per student-week), but achieved considerably lower control (8% vs 21% reduction of peak incidence). The reactive class closure implemented in Italy was predicted to be very costly, leading to > 20% student-days lost.

**Conclusions:**

At high incidence conditions, reactive screening protocols generate a large and unplanned demand in testing resources, for marginal control of school transmissions. Comparable or lower resources could be more efficiently used through weekly screening. Our findings can help define incidence levels triggering school protocols and optimise their cost-effectiveness.

**Key Public Health message**

**What did you want to address in this study?**

During January 2022, the high incidence of SARS-CoV-2 Omicron variant infections led to school disruptions in many European countries. Authorities thus implemented protocols to lessen the virus spread in school settings. We wished to understand which of three widely used protocols, performed best, considering test demand, infection prevention, and school absences. The protocols included ‘reactive screening’, ‘weekly screening’ and ‘reactive class closure’.

**What have we learnt from this study?**

When incidence of SARS-CoV-2 infections is high, school protocols based on reactive screening lead to a substantial and unplanned demand for testing resources, while little infection prevention is achieved. With the same resources, proactive weekly screening considerably reduces the peak of infections, limiting schooldays lost. Reactive class closure leads to large disruption with successive closures.

**What are the implications of your findings for public health?**

Our findings provide key information to improve prevention and control strategies of SARS-CoV-2 transmission in the school setting. They can be used to tune the response by defining incidence levels triggering school protocols, depending on the severity of the circulating variant and according to the objectives established by authorities.

## Introduction

At the start of 2022, countries in Europe faced large disruptions in schools due to the exceptionally high incidence rates of the severe acute respiratory coronavirus 2 (SARS-CoV-2) Omicron variant (Phylogenetic Assignment of Named Global Outbreak (Pango) lineage designation: B.1.1.529) [1]. As the adult population was largely covered by vaccination, higher incidence rates were reported for the first time in children and adolescents compared with other age groups [2]. In France, nearly 7,000 coronavirus disease (COVID-19) cases per 100,000 were reported among 6–10-year-old and 11–19-year-old individuals at the peak of the Omicron wave in January 2022, compared with ca 4,500 cases per 100,000 among the 20–59-year-olds [3]. Despite protocols implemented by national authorities to ensure in-person attendance in schools, school establishments were nevertheless put under pressure by the high incidence rates. Protocols required repeated quarantines, disrupting attendance and learning, or led to large and sudden testing demands for children, overloading saturated surveillance systems [4,5].

Through modelling, here we compared the school protocols adopted by France, Switzerland, and Italy, in terms of resource peak demands, infection prevention, and reduction of schooldays lost, under the high incidence conditions experienced in January 2022 during the Omicron BA.1 variant wave.

## Methods

### Modelling SARS-CoV-2 transmission in schools

We adapted to the Omicron wave a stochastic agent-based model of SARS-CoV-2 transmission at school presented in detail by Colosi et al. [6]. The model uses empirical data on time-resolved face-to-face proximity contacts between individuals in a primary school in France, collected using wearable radio frequency identification (RFID) sensors [7]. The dataset includes 232 students (aged 6–10 years) and 10 teachers organised in 10 classes, two classes per grade. Students were found to spend on average more time interacting with other students of the same class than across classes, and to establish longer contacts compared with teachers [6]. We described SARS-CoV-2 infection progression through the following disease stages: latency, prodromic stage, clinical and subclinical stages, recovery from infection (Supplement 1.1. Compartmental model and parameters). Stages were informed from empirical distributions, and accounted for age-specific estimates of susceptibility, transmissibility, probability of developing symptoms, and probability to detect a case based on symptoms [8-18].

We modelled the circulation of the Omicron variant, considering 20% protection after infection from prior variants [19], an intrinsic transmissibility advantage of 30% relative to the Delta variant (Pango: B.1.617.2) [20], and a shorter incubation period of 0.5 days compared to the Delta variant [20,21]. Omicron’s higher spreading rate was considered to be mainly due to immune evasion [20], in line with observations from household studies [22], but we also tested a transmissibility advantage of 80% relative to Delta for sensitivity (Supplemental Table S1. Parameters, values and sources used to define the compartmental model). The transmissibility advantage was applied to the within-school transmissibility of previously circulating variants that we inferred in prior work from observed prevalence in French schools [6]. We calibrated the model to reproduce the reported community surveillance incidence in primary school students (6–10 years old) in France in January 2022 [3], and considered additional scenarios of Omicron waves reaching lower and higher peaks to capture the variability of the wave across European countries [2]. Additional details are provided in the online Supplement (section 3.1. Incidence and number of tests per student over time under different introduction conditions).

### School protocols

We modelled the school protocols adopted in France, in the Baselland canton in Switzerland, and in Italy. We simulated the reactive protocol applied in France in January 2022, requesting an anterior nasal lateral flow device (LFD) test at days D0, D2, and D4 to the class of the detected case, following case identification [23]. Students with positive tests had to isolate for 7 days. For sensitivity, we tested reactive screening with different numbers and lags for control (D0, D3, D7 and D0, D4, see Supplement 4.5. Sensitivity analysis on control screening). In Baselland, students were tested on a voluntary basis every week with salivary PCR tests [24]. We thus simulated a regular screening strategy, considering two options for the frequency of screening, once a week (as in Baselland), and twice a week, with a 75% adherence of the school population (min–max range of 50–100%). Regular screening was performed on all participating individuals, regardless of the presence of symptoms. Students with positive tests were isolated for 7 days. Finally, we simulated the reactive class closure adopted in Italy, requiring a quarantine of 10 days for the students of the class of the detected case [25]. These protocols were considered independently in the analysis, as each corresponded to a national strategy. In all cases, we also considered symptomatic testing and case isolation.

The model was informed with time-varying and age-dependent test sensitivity, yielding an estimated 67% peak sensitivity for asymptomatic children in nasal LFD tests and 96% in salivary PCR tests [26] (Supplement 1.5. Parameters for screening and testing protocols). We also explored a lower peak sensitivity of 55% for LFD tests.

### Vaccination

The model was further stratified to account for vaccination status and to include vaccine effectiveness (VE) against infection and transmission (Supplement 1.6. Vaccination). By the first week of January 2022, 94% of adults (18–59 years) in France were vaccinated with at least two doses, and 45% had received the third dose since the opening of the vaccination campaign on 27 November 2021 [27]. We therefore considered in the model that all teachers completed the primary vaccination, with 50% of them having received also the third dose, i.e. the booster. As adults were recently boosted, we considered the following values for the VE against infection: a VE of 70% for teachers vaccinated with three doses, corresponding to the estimate within the first 4 weeks since the third dose [28]; a VE of 15% for those with two doses only, corresponding to the estimated waned efficacy at 6 months after the second dose [28]. For sensitivity, we varied the booster vaccination coverage in teachers up to 100% (Supplement 4.1. Sensitivity analysis on vaccination coverage for teachers).

The vaccination campaign in children (5–11 years) opened on 22 December 2021 [29]. By mid-January 2022, the coverage in this age group in France was < 3% [3], therefore we assumed no vaccinated children in the main analysis. We then tested higher vaccination coverages (20%, 40%, 60%) in children in the scenario analyses, considering high (VE = 50%, estimated within the first 4 weeks from vaccination [30]) and low (VE = 20%) values of VE against infection.

## Results

**Figure 1:**
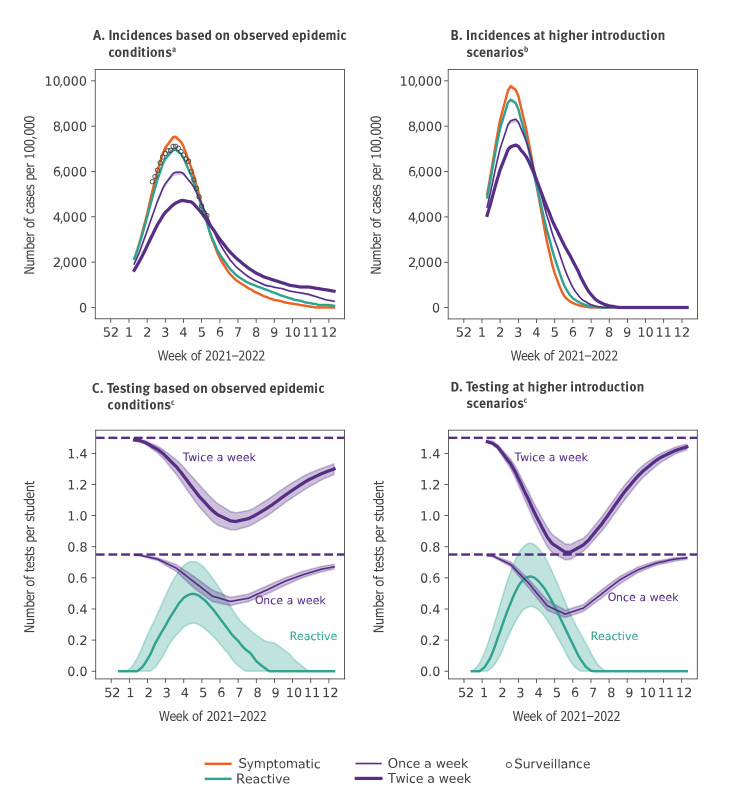
Incidence of COVID-19 cases among 6-10-year-olds and number of tests per student over time under different school protocols, France, January 2022

COVID-19: coronavirus disease; IQR: interquartile range; LFD: lateral flow device.

^a^ Simulated weekly incidence expressed in number of COVID-19 cases in students per 100,000 over time for different protocols, and reported incidence in the 6–10 year age group in France in the period 10 Jan–6 Feb 2022 [3]. The reactive protocol, applied in France, is calibrated to surveillance data.

^b^ As in A for simulated scenarios at higher introduction conditions.

^c^ Panels C,D: average number of tests per student over time for reactive and regular protocols under the epidemic conditions illustrated in panels A and B respectively. The horizontal dashed lines indicate the theoretical values of the demands in number of tests per student, in the screening once a week and twice a week (i.e. imposed by 75% adherence and by the frequency). Results are obtained considering the use of nasal LFD tests in both reactive and regular screenings. Shaded areas around the curves correspond to the IQR.

Simulations capture well the reported dynamics of community surveillance incidence in primary school students (6–10 years) in France (**Figure 1A**). The reactive protocol implemented by authorities was predicted to marginally reduce the peak, whereas regular screening would flatten more substantially the curve. The median number of tests required by the reactive protocol increased along the wave, with a predicted peak demand of 0.50 (interquartile range (IQR): 0.32 to 0.71) tests per student per week at the incidence rate experienced in France (ca 7,000 cases per 100,000 among 6–10-year-olds; **Figure 1C**). Test demand instead was predicted to decrease in the regular protocols because fewer students would be present in class after the peak of infections due to isolation, with 0.45 (IQR: 0.42 to 0.47) tests in the once-a-week screening and 0.96 (IQR: 0.91 to 1.02) in the twice-a-week screening. We found that higher incidence conditions could lead to a larger demand of tests by the reactive protocol compared with the weekly screening (**Figure 1B,D**).

**Figure 2:**
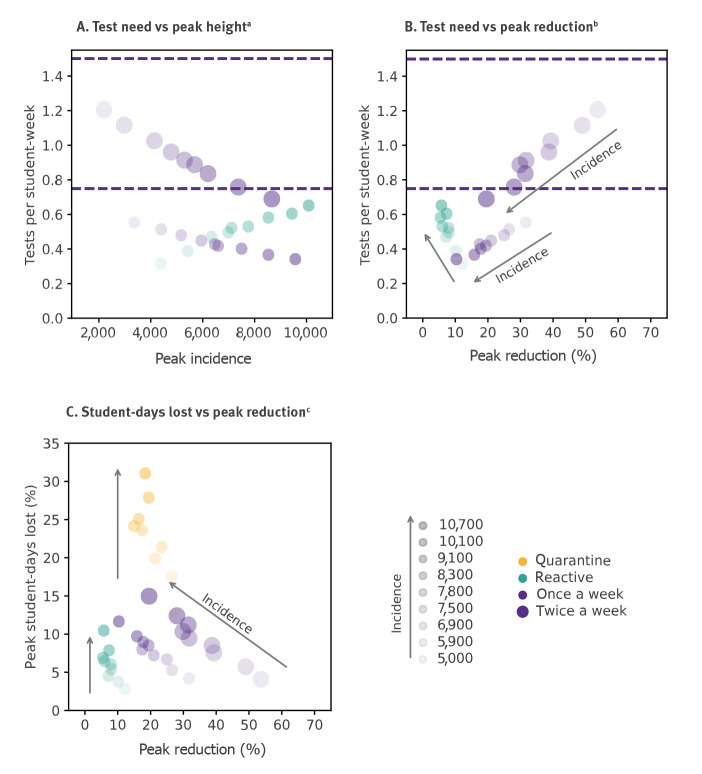
Test needs and schooldays lost vs peak reduction at varying peak incidence rates

To evaluate how to best use resources, we estimated the impact of protocols in reducing the peak incidence and extended the analysis of **Figure 1** to a larger set of Omicron wave scenarios with varying peak incidence. For the incidence rates registered in France in January 2022, reactive screening was estimated to lower the peak by 8% (IQR: −3% to 19%), compared with 21% (IQR: 11% to 31%) reduction achieved by the weekly screening (**Figure 2A,B**), despite the higher demand in testing resources at the peak (0.50 vs 0.45 tests per student-week, respectively).

COVID-19: coronavirus disease.

^a^ Demand in the number of tests per student-week at peak as a function of the peak incidence (COVID-19 cases in students per 100,000) for the reactive protocol and the regular screening protocols with once a week and twice a week frequency. The horizontal dashed lines indicate the theoretical values of the number of tests per student in the regular screening (i.e. imposed by 75% adherence and the frequency). Dots reduce their transparency for increasing incidence.

^b^ Demand in the number of tests per student-week at peak as a function of the percentage of peak reduction achieved by each protocol compared with symptomatic testing (i.e. in absence of interventions), for different incidence levels. The horizontal dashed lines are as in panel A. Dots transparency code is the same as in panel A.

^c^ Peak percentage of student-days lost as a function of the percentage of peak reduction achieved by each protocol compared with symptomatic testing, for different incidence levels. The reactive quarantine of the class is shown as an additional protocol. Arrows are shown as a visual guide.

The predicted number of tests required by the reactive screening would increase for increasing values of the incidence rate (from 0.31 to 0.65 tests per student-week corresponding to 5,000 to 10,700 cases per 100,000), but they would achieve a marginal control of the viral circulation at school, reducing the peak of the wave by at most 12%. Results would not change by changing the lags of the reactive screening (D0, D3, D7 vs D0, D2, D4) but peak reduction would be even lower if a lower number of screenings was adopted (D0, D4; Supplement 4.5. Sensitivity analysis on control screening). Regular screening would instead achieve 20% or more of peak reduction for incidence rates up to 7,500 cases per 100,000 with a weekly frequency, and for rates up to 10,100 cases per 100,000 if screening the school twice a week. Similar results were obtained considering the reduction of the epidemic size of the full wave and a higher transmissibility advantage of the Omicron variant (Supplement 3.2. Test needs and schooldays lost vs. percentage of case reduction at varying incidence rate and, Supplement 4.2. Sensitivity analysis on advantage in transmission rate of Omicron relative to Delta).

Student-days lost remained below 12% with reactive and weekly screening, whereas reactively closing the class as in the Italian protocol could lead to > 20% of absence per student if peak incidence is over 7,500 cases per 100,000 (**Figure 2C**). Findings were robust against changes in booster coverage in teachers, in Omicron transmissibility and incubation period (Supplement 4.1, 4.2, 4.3). Higher detection rates would penalise the reactive screening, due to an increase in test demand while control would remain limited (Supplement 4.4).

**Figure 3:**
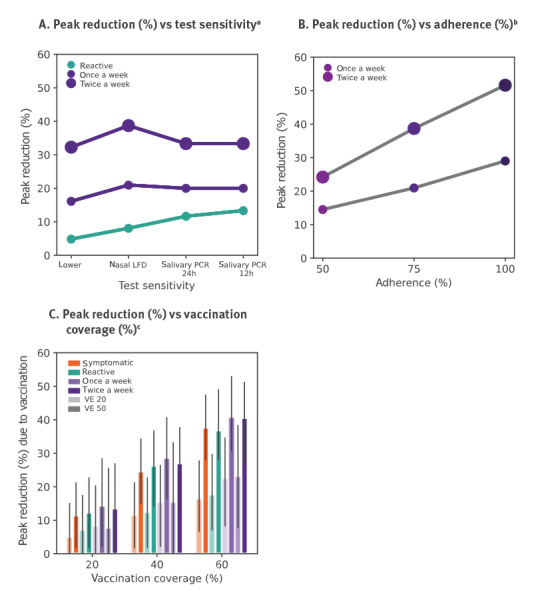
Impact of test sensitivity, adherence to regular screening, and vaccination

LFD: lateral flow device; Pango: Phylogenetic Assignment of Named Global Outbreak lineage designation; VE: vaccine effectiveness.

^a^ Percentage of peak reduction achieved by each protocol compared with symptomatic testing (i.e. in absence of interventions) as a function of the test sensitivity and of the delay in returning the results (12 h from testing,  24 h from testing for PCR tests). The lower value corresponds to 55% peak sensitivity.

^b^ Percentage of peak reduction as a function of adherence to regular screening.

^c^ Percentage reduction in the peak incidence for each protocol due to vaccination in children, for different vaccination coverages at respective values of VE of 50% (solid bars) and 20% (transparent bars).

Changing from nasal LFD tests to salivary PCR tests would improve the reactive strategy from 8% to 13% peak reduction if results were available after 12 h (**Figure 3A**). Instead, regular testing was predicted to be mainly affected by adherence to screening (**Figure 3B**). Vaccinating 6–10 years old children was predicted to provide a collective benefit in reducing viral circulation at school. If children were vaccinated close to the epidemic wave (therefore with an estimated VE of 50% for children within 4 weeks after the second dose), the peak would be reduced by ca 30% for 40% coverage and by ca 40% for 60% coverage, compared with no vaccination (**Figure 3C**). If vaccination occurred long before the epidemic wave (waned vaccine effectiveness VE = 20%), the reductions would be smaller, around 15% and 20% for 40% and 60% coverage, respectively.

Results of all panels refer to the Omicron (Pango lineage designation: B.1.1.529) wave shown in Figure 1A.

## Discussion

For the high incidence rates recorded in January 2022 in Europe due to the Omicron BA.1 variant, our study predicted that reactive screening strategies in schools, as employed in France, required a higher number of tests per student per week compared with weekly screening, but achieved a lower epidemic control. The protocol requesting three tests in less than a week for case contacts in French primary schools led to large disruption events in January 2022, in terms of logistics, resources, and impact on surveillance capacity [4]. We estimated that the same resources would have been more efficiently used by weekly screening schools, reaching 21% peak reduction for the incidence rates registered in France in January 2022, compared with the marginal reduction (8%) estimated for the reactive screening.

Reactive screening is predicted to be poorly effective in case prevention for two main reasons. First, timely interventions of case identification and isolation are key to control SARS-CoV-2 spread, given the presence of pre-symptomatic and subclinical transmission [31]. Reactive strategies suffer from considerable time lags compared with the ongoing transmission dynamics. Second, this aspect is particularly challenging in children as they have a lower probability of developing symptoms [12,13,15,17,32], and therefore of being identified as possible COVID-19 cases compared with adults. By the time the screening is activated, after the detection of a case based on recognisable symptoms, the transmission may have already occurred in the school and have previously generated asymptomatic infections that went unnoticed. That is, the case triggering the screening does not necessarily represent the start of the chain of transmission and may instead occur after few generations of cases that are not discovered by surveillance, or following undetected introductions. On the contrary, regularly screening the school every week or twice a week allows the prompt identification and isolation of infectious individuals regardless of their symptoms [6,32-39]. As more cases are found, onward transmissions are more efficiently prevented, with a higher efficiency if frequency of screening is higher. The capacity of screening (whether reactive or proactive) to reduce the peak incidence decreases for increasing values of the incidence rates. Higher incidence in the community indeed corresponds to larger rates of introductions in the school, which require an even more rapid response for the identification of cases to prevent onward transmission [6].

Some countries opted for systematically screening schools against SARS-CoV-2 transmission, supported by numerical evidence [6,32-39]. Authorities in Baselland (Switzerland) offered weekly salivary PCR tests to all schools since March 2021. Prior to making participation mandatory in 2022, recorded adherence was on average rather high (> 75%) [24]. Proactively screening also has the advantage of planning resources in advance, contrary to reactive screening subject to sudden peak demands and potential shortages. This was reported to help simplifying the logistics of test-to-stay strategies in pilot weekly screenings implemented in a number of pre-primary and primary schools in the Auvergne-Rhône-Alpes region in France in December 2021. Preliminary unpublished empirical estimates from these screenings also suggest a reduction of cases during the Delta wave in December 2021 compared with the reactive strategy, in line with model predictions.

The widespread access to nasal antigenic tests made repeated self-testing possible without loss in efficiency, as lower sensitivity is compensated by promptness of results and high frequency [40]. Regular self-testing would also limit the high rates of absence from school that are associated to reactive class closures. Without test confirmation, reactively closing the class imposes the quarantine to likely uninfected students who would unnecessarily miss school while transmission may have already occurred in other classes due to cross-classes contacts or through introductions [6]. Under the high incidence rates registered in the Omicron wave, our model predicted multiple class closures continuously disrupting the school rhythm and impacting students’ learning, with more than 20% of schooldays lost per student, compatible with observations in Italy during that wave [5].

This study focused exclusively on the school setting, and did not assess the impact that protocols at schools, aimed at limiting school transmission, may have on the epidemic dynamics in the community. Model-based findings previously highlighted that protocols mitigating viral circulation at school also reduce the spread in the community [34,41,42]. Conversely, increased transmission in the community was found to be associated to schools in session [43,44], and households with children were estimated to be at higher risk of SARS-CoV-2 infection [45], suggesting that a considerable fraction of transmission events originated from the school setting [46]. The analysis of a school outbreak in early 2021 in a municipality in the north of Italy estimated that ca 21% of SARS-CoV-2 transmissions were associated with school contacts, compared with 50% and 29% transmissions associated with household and community contacts, respectively [47]. Combined with the above evidence, our findings therefore suggest that implementing strategies to control transmission at school will reduce the potential for seeding transmissions from schools to other settings, narrowing the spread across households [48] and the risk of reaching individuals at risk of COVID-19 complications.

Our findings can be used to tune the response by defining incidence levels triggering protocols if facing a high incidence wave, depending on the severity of the circulating variant and according to the objectives established by authorities. Systematically screening schools remains the optimal test-to-stay strategy, reducing peak incidence rates in children, and thus their consequences on hospitalisations [49] and long COVID [50] in this age group, while limiting school disruption and requested resources. Large vaccination coverage in children contributes to mitigate high viral circulation, making schools safer. Coverage remains, however, low in children in several European countries (16% median coverage for 2-dose vaccination in 5–9 years old by the start of September 2022; Supplement S9. Vaccination coverage of children in Europe).

Our study has limitations. We did not consider immunity waning over time as we focused on a single pandemic wave, but tested low vaccine effectiveness to account for the estimated reduction associated with the lag from the last vaccination dose. Our results are framed within the context experienced by European countries. As such, results are not directly applicable to other countries with a context of lower population immunity due to the limited spread of earlier variants. In previous work, however, we showed that conclusions are qualitatively robust, with regular screening strategies outperforming reactive strategies in case prevention under a set of different epidemic and immunisation conditions [6].

A large demand in tests results from reactively screening schools in high incidence conditions. Comparable resources could be more efficiently used in a proactive screening strategy to mitigate the peak.

## Supplementary Data

1318000 Supplementary Material
