# Supplementary material for: Minimising school disruption under high incidence conditions due to the Omicron variant in France, Switzerland, Italy, in January 2022
Source: Euro Surveill. 2023 Feb 2;28(5):2200192. doi: 10.2807/1560-7917.ES.2023.28.5.2200192 (PMC9896604; doi:10.2807/1560-7917.ES.2023.28.5.2200192)
Supplement: Supplementary Material [file 2200192_SupplementaryMaterial.pdf]

SUPPLEMENTARY INFORMATION

Minimising school disruption under high incidence conditions due to the Omicron variant in France, Switzerland, Italy in January 2022

"This supplementary material is hosted by Eurosurveillance as supporting information alongside the article Minimizing school disruption under high incidence conditions due to the Omicron variant in France, Switzerland, Italy in January 2022, on behalf of the authors, who remain responsible for the accuracy and appropriateness of the content. The same standards for ethics, copyright, attributions and permissions as for the article apply. Supplements are not edited by Eurosurveillance and the journal is not responsible for the maintenance of any links or email addresses provided therein."

Contents

1. Transmission model 2
1.1. Compartmental model and parameters 2
1.2. Empirical contact patterns 4
1.3. Within-school transmission 5
1.4. Modelling transmission outside schools: introductions conditions 5
1.5. Parameters for screening and testing protocols 6
1.6. Vaccination 9
2. Inference framework 11
3. Supplementary results 11
3.1. Incidence and number of tests per student over time under different introduction conditions 11
3.2. Test needs and schooldays lost vs. percentage of case reduction at varying incidence rates. 12
3.3. Vaccination coverage for children 12
4. Sensitivity analysis 13
4.1. Sensitivity analysis on vaccination coverage for teachers 13
4.2. Sensitivity analysis on advantage in transmission rate of Omicron relative to Delta 13
4.3. Sensitivity analysis on latent period of Omicron 14
4.4. Sensitivity analysis on detection probability 14
4.5. Sensitivity analysis on control screening 15
5. References 15

1. Transmission model

1.1. Compartmental model and parameters

The progression of the infection for each host in the agent-based model follows the structure illustrated in **Figure S1**. Susceptible individuals (S) and individuals who were previously infected by other variants but are susceptible to the Omicron variant (S^*) become exposed (E) following infection. After a latent period, exposed individuals enter a prodromic phase (I_p) during which they can transmit the disease without showing symptoms. Following that phase, individuals can develop a subclinical infection (I_{sc}) with probability p_{sc}, otherwise they develop clinical symptoms (I_c). We considered individuals in the prodromic and subclinical compartments to be less infectious than clinical individuals, and to remain undocumented unless tested [1–4]. After the infectious phase, individuals enter the recovery state (R_+, R), where they become immune and no longer transmit the infection. This state is divided into two successive states, R_+ and R, to isolate a first period (R_+) during which the individual is no longer infectious but PCR tests may still detect the presence of the virus in the upper respiratory tract [5]. We assumed the protection against infection from prior variants to be 20% [6].

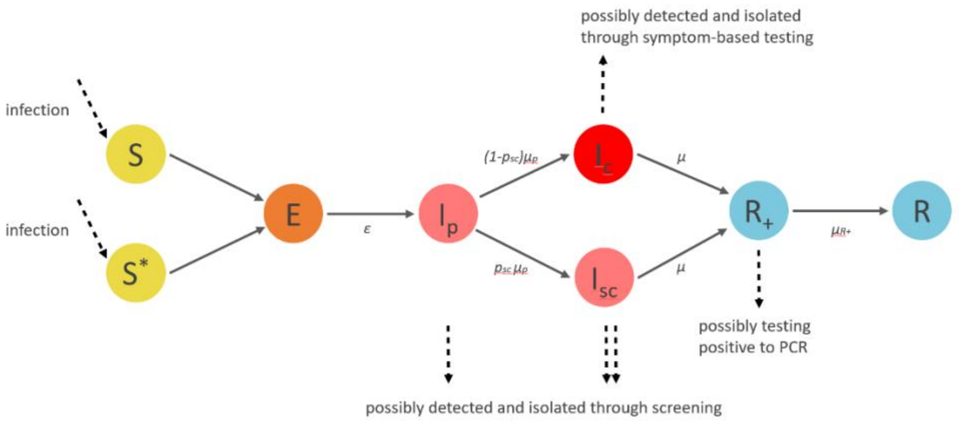

Figure S1. Structure of the compartmental model.

Structure of the compartmental model.

The E, I_p, I_c, I_{sc} compartments are further split into sub-compartments to capture a non-exponentially distributed duration of stay in each infection stage, following empirical data [7–16].

The compartmental model is informed with empirical distributions of disease state periods. We modified the distributions specific to the Delta variant [17] (**Figure S2**) to account for the shorter latent period of the Omicron variant [18,19]. In the main analysis we considered a period shorter of 0.5 days, and for sensitivity we tested a period shorter of one day.

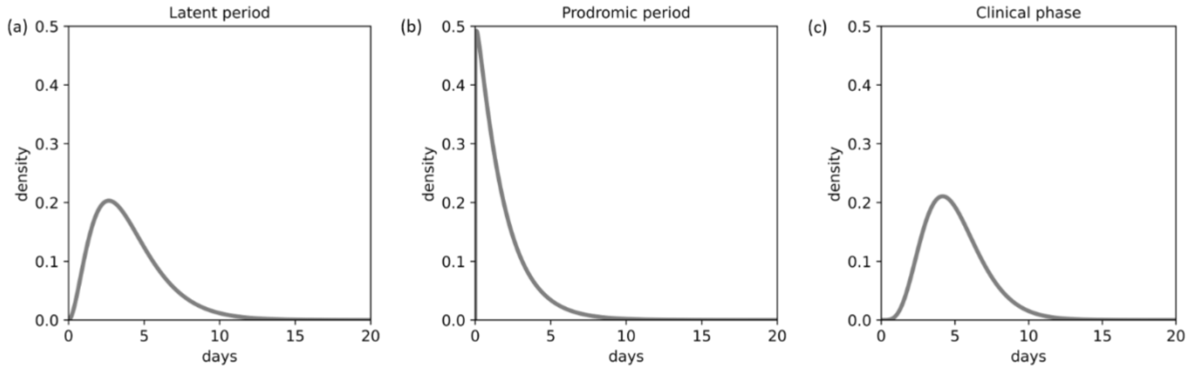

Figure S2. Distributions of disease state periods of the Delta variant. (a) Gamma distributed latent period with mean=4.00 days and std=2.30 days (shape=3.00, scale=1.33); (b) Gamma distributed prodromic period with mean=1.80 days and std=1.75 days (shape=1.05, scale=1.71); (c) Gamma distributed clinical phase with mean=5.00 days and std=2.03 days (shape=6.00, scale=0.833). Distributions of disease state durations are assumed to be the same for students and teachers. Estimates from [17].

Table S1 summarizes the parameters of the compartmental model and their values. The compartmental model is further stratified to consider vaccination in the population (subsection 1.6).

Table S1. Parameters, values, and sources used to define the compartmental model.

Variable	Description	Value	Source
σ	Relative susceptibility to infection compared to adults	50%	[20–22]
p_{sc}	Probability of subclinical infection	0.8 for students<br>0.5 for teachers	[4,23–26]
β	Transmissibility per contact per unit time	+30%, +80% compared to the Delta variant	[18,27]
r_β	Relative transmissibility in prodromic and subclinical stages	55% if I_p, I_{sc}	[1–4]
$r_\beta^{children}$	Relative transmissibility of children	63%	[28]
--	Protection against infection from prior variants	20%	[6]

The model is initialized with 40% natural immunity from seroprevalence estimates [29] and accounting for Delta infections during the start of the fifth wave in France, before Omicron became dominant. Omicron infections are generated by the simulations. Vaccination status is based on data (subsection 1.6).

To parametrize the Omicron wave scenarios, we used the per-contact transmissibility β^{Delta} previously estimated for the Delta variant in [27] and considered the transmissibility advantage of Omicron compared to Delta [18]. We considered a transmissibility advantage (expressed in multiplicative form) ϕ between 30% and

80% of Omicron compared to Delta [18]. We set ϕ =30% in the main analyses and explored ϕ =80% for sensitivity.

1.2. Empirical contact patterns

We used empirical data describing time-resolved face-to-face proximity contacts between individuals collected in a primary school in France using wearable RFID sensors in a pre-pandemic period. The dataset describes the contacts among 232 students (6-11 years old) and 10 teachers in a primary school in Lyon, composed of 5 grades, each of two classes [30].

We built temporal contact networks, composed of nodes representing individuals (classified by class and student/teacher), and links representing empirically measured proximity contacts occurring at a given time (**Figure S3**). As the dataset covers only two days, we developed an approach to temporally extend the datasets by generating synthetic networks of contacts that reproduce the main features observed empirically (class structure, within- vs. between-classes links, contact duration heterogeneity, and similarity across days). In **Figure S4** and **Figure S5** we show that synthetic networks well reproduce the heterogeneity in contact duration between pairs of individuals and the timeline of activity, respectively. More details are provided in Ref. [27].

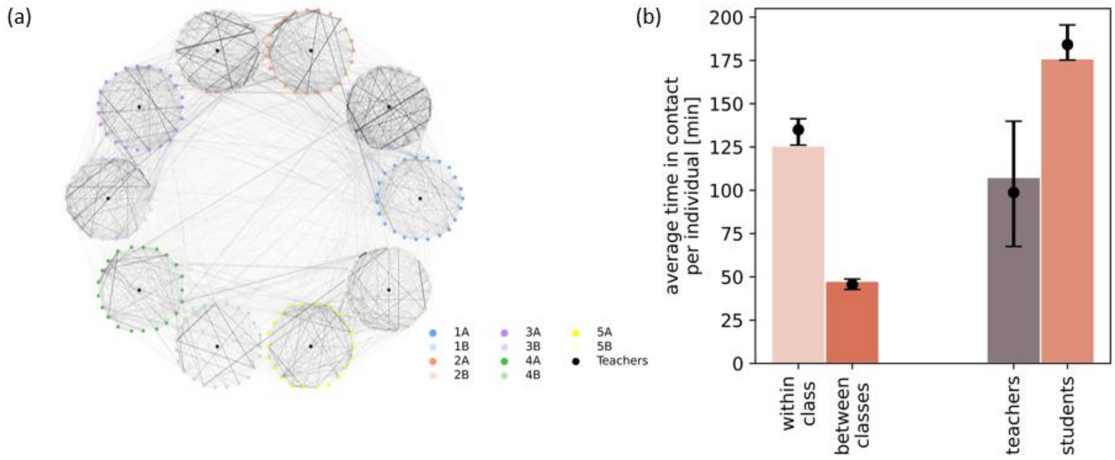

Figure S3. Empirical contact networks. (a): Empirical temporal contact data aggregated over two days. Nodes represent teachers and students, circles classes (different colours), and links contacts (thickness coding duration). (b): Daily average time that an individual spends in interaction within the same class or in different classes (left), and daily average contact time of teachers and students (right). Bars refer to empirical networks, points and errors (95% bootstrap confidence intervals) to synthetic networks.

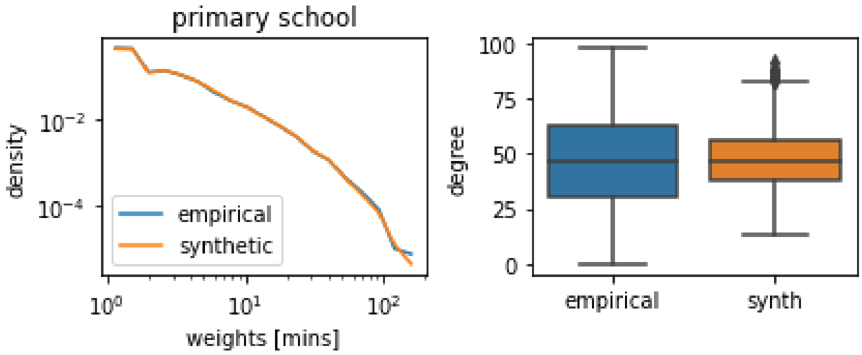

Figure S4. Distributions of daily contact durations (left) and degree distributions (right) for the empirical and synthetic contact networks. For the degree distributions, the boxplot central horizontal line gives the median value of the distribution, the box shows the quartiles of the data sets, the whiskers extend to show the rest of the distributions, except for outliers defined as being more than 1.5IQR above the upper quartile.

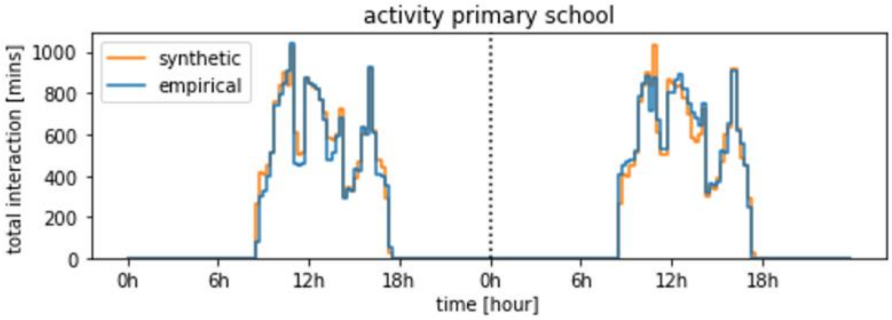

Figure S5. Timelines of contact durations for the empirical and synthetic data. The line gives the value of the total time in contact of all individuals during 15-minutes time windows.

1.3. Within-school transmission

Within-school transmission is modelled with an algorithm based on rejection sampling [31]. In the absence of testing protocols, it proceeds as follows.

At each time step Δt of 15 minutes, the pairwise interactions described in the contact network are evaluated. If a contact of duration $w \cdot \Delta t$ happens between an infectious (I_p, I_{sc}, I_c) and a susceptible (S) individual in the time step Δt, the susceptible individual may become exposed with a probability p given by

$$p = \beta \cdot \Delta t \cdot w \cdot \sigma_S \cdot r_\beta$$

where β is the transmission rate, σ_S is the susceptibility (which depends on age), r_β is the relative transmissibility (which depends on both age and infectious state). In particular, p is proportional to the duration of the interaction recorded in the contact network ($w. \Delta t$), and to the age-specific epidemiological features of the individuals (σ_S, r_β).

Then, spontaneous transitions between successive compartments are evaluated. The time spent in each compartment follows the distributions of **Figure S2**, modified to account for a shorter latent period of the Omicron variant compared to the Delta.

School days include Monday, Tuesday, Thursday and Friday. The simulations run for a total of 90 days, with the distinction between school days and non-school days. In the latter and during off-school hours, only spontaneous transitions are evaluated.

An individual may become exposed (E) due to community interactions outside the school, thus we included a weekly number of introductions due to out-of-school infections (as explained in the next subsection).

1.4. Modelling transmission outside schools: introductions conditions

Contacts outside the school are not explicitly modelled, as the sensor data we use capture interactions within the school setting only. Outside-school contacts (i.e., contacts between individuals of the school population and their family and friends, as well as their casual contacts in the community) are responsible for the introduction of infected individuals in the school, as teachers and students can get infected while in contact with the rest of the community.

Weekly introductions at school were modelled stochastically considering a plausible range of age-specific community incidence, to capture the situations experienced across countries in Europe. For a weekly incidence i_{age}, the prevalence p_{age} can be estimated as $p_{age} = i_{age}/p_{d,age}$, accounting for the probability of detection $p_{d,age}$ and considering 7 days of infectious period [32,33]. The expected number of COVID-19 introductions in

a specific age class of size n_{age} (n=232 students, n=10 teachers) can then be modelled with a binomial distribution:

$$Intro_{age}(w) \sim B(n_{age}, p_{age}). \quad (1)$$

As we are interested in modelling Omicron epidemic waves of different peak incidence levels (corresponding, for example, to country-specific incidence rates), we explored average weekly introductions ranging from about 0.8 to 23 per week, corresponding to initial incidence levels in the range 100-3,000 per 100,000. These conditions correspond to peak incidence rates between 4,000 and 10,100 cases per 100,000, in absence of interventions. Results are evaluated with respect to peak incidence rates.

Results of Figure 1A of the main text are obtained from fitting the model to the observed incidence rates in 6-10 years old children to infer the weekly introductions (see Section 2).

1.5. Parameters for screening and testing protocols

We considered empirically estimated test sensitivity over time. Different estimates exist on the relative performance of RT-PCR tests on saliva with respect to naso-pharyngeal samples [34,35], but evidence suggests that the performance of saliva samples is as good as that of naso-pharyngeal samples for symptomatic individuals [36–52]. For asymptomatic individuals, a similar relation is recovered during the early stages of the infection, but evidence suggests that saliva samples may be less sensitive than naso-pharyngeal swabs for detecting the virus during convalescence [53,54].

For these reasons, for test sensitivity on RT-PCR saliva samples, we considered:

- the time-varying test sensitivity of RT-PCR naso-pharyngeal samples from symptomatic cases [55], [56], interpolating data before the onset of symptoms with data after the onset of symptoms [55,56] (**Figure S6a**). The peak value of sensitivity is 96% [55].
- the time-varying test sensitivity of RT-PCR saliva samples from asymptomatic cases [57] (**Figure S6b**). Sensitivity values are defined in terms of days since the first matched positive viral culture from an anterior nasal sample, commonly used to align the time axis to symptom onset [13,58].

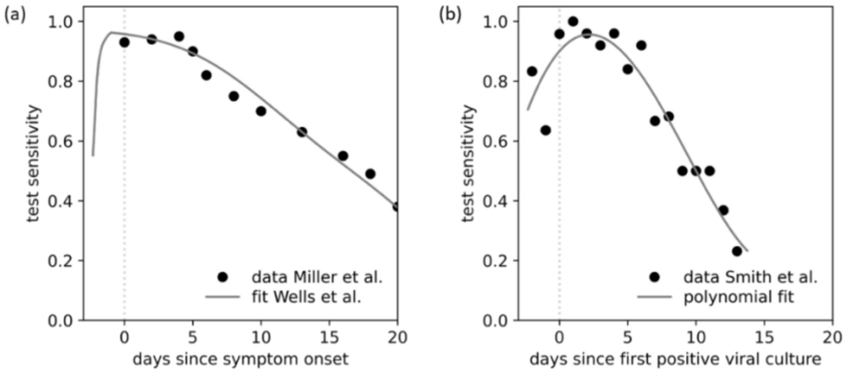

Figure S6. Time-varying test sensitivity of RT-PCR naso-pharyngeal and RT-PCR saliva samples. (a) Samples from symptomatic cases, data from [56] and fit from [55]. (b) samples from asymptomatic cases. Data from [57] were fitted with a polynomial of degree 4 minimizing the mean squared error.

For the time-varying sensitivity of LFD tests, we used data obtained from samples collected in asymptomatic adults for which data over time are available [57] (**Figure S7a**). Sensitivity was shown to vary between asymptomatic and symptomatic cases, and between age classes [59–63]. In absence of time-varying data for all these stratifications, we assumed the time dependence from [57] and rescaled it to match the peak sensitivity values for symptomatic and asymptomatic cases in adults and children obtained from Ref. [64]. Peak values are in accordance with the literature [60,65,66].

Sensitivity over time was then adjusted to the sensitivity of the RT-PCR anterior nasal tests, as the sensitivity of the LFD tests was measured with respect to the latter [57,64]. We fixed the sensitivity of the RT-PCR anterior nasal tests at 88%, the upper bound of the estimate provided by the review of Ref. [67] relative to the

sensitivity of the RT-PCR naso-pharyngeal test, in agreement with other estimates [42,68–72]. Once adjusted, the peak value of the absolute sensitivity of the LFD test for a symptomatic adult was 81%, considering the peak value of the RT-PCR naso-pharyngeal samples being equal to 96% [55]. Results are shown in **Figure S7b**.

Finally, we explored a lower peak sensitivity of 55% for asymptomatic children, for both test types (**Figure S7c**).

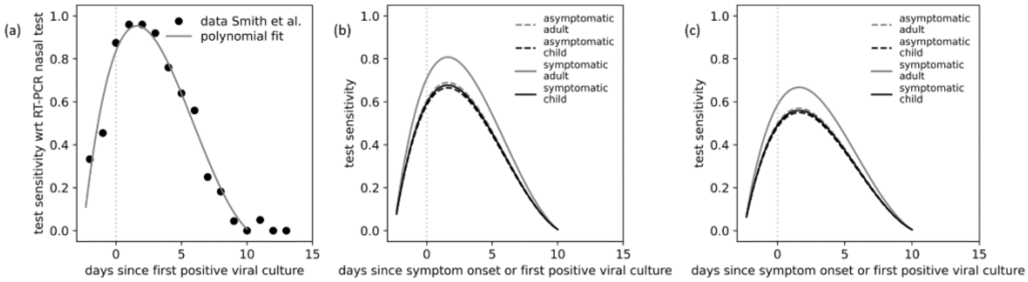

Figure S7. Time-varying test sensitivity of lateral flow device nasal antigen tests. (a) Test sensitivity is relative to the test sensitivity of PCR nasal tests, from adult asymptomatic cases [57]. The peak value of sensitivity is 95%. Data from [57] was fitted with a polynomial of degree 4 minimizing the mean squared error. (b) Absolute test sensitivity considering the sensitivity of the RT-PCR anterior nasal tests at 88% [67] and the sensitivity of the RT-PCR nasopharyngeal test 96% [55]. The time dependence measured in [57] is adjusted to match the peak sensitivity values from [64] for symptomatic and asymptomatic cases in adults and children. (c) Absolute test sensitivity considering the peak sensitivity of the LDF test for asymptomatic children at 55%. Value explored in the absence of empirical estimates on test sensitivity against Omicron infection.

The probability of detection of COVID-19 infection based on symptoms is age-dependent and informed from the literature [73,74] (**Table S3**). We further explored a range for sensitivity analysis.

All detected cases (identified on the basis of their symptoms or following a screening) are put in isolation for a duration of 7 days. The only exception is the Italian protocol of reactive class closure which required 10 days of isolation for cases and students in their class. At the end of the isolation period, students return to school without further testing, following current recommendations. We consider that teachers are required to show a negative PCR test before returning to school, accounting that the virus can still be detectable by a PCR test after the infectious period [5]. In our model, every time a teacher is detected, she is replaced by a susceptible substitute.

We considered a frequency for regular screening of once per week, and twice per week. We explored adherence in the range from 50% to 100%. We considered a turnaround time of 15 minutes for LFD tests, and of 12h and 24h for PCR tests on saliva samples. We fixed test specificity to 100% for both tests [75].

Table S3 summarizes the parameters and values used in the model.

Table S3. Parameters, values, and sources used to define detection and isolation in testing and screening protocols.

Variable	Description	Value	Source	Values explored for sensitivity
p_d	Probability of detection of COVID-19 infection among clinical cases	0.3 for primary school students<br>0.5 for teachers	[73,74]	0.2, 0.5
Δq	Duration of isolation and reactive quarantine	7 d, 10 d	7d: Informed by the protocol of the French Ministry of Education<br><br>10d for reactive class closure only: Informed by the protocol of the Italian Ministry of Education [76]	
W	Weekly frequency of the regular screening	1, 2 per week	Weekly frequency as in protocol adopted in Baselland canton, Switzerland.	
f	Adherence to regular screening	75%	From recorded adherence in weekly screening in Baselland canton.	50%, 100%

1.6. Vaccination

The compartmental model illustrated in **Figure S1** is further stratified to consider vaccination in the population, as shown in **Figure S8**. As we are interested in assessing the role of vaccination coverage in the school population within a relatively short timeframe, we do not consider a dynamic vaccination rollout, and assume that vaccination coverage is fixed throughout the simulation. The impact of different coverage levels is assessed by exploring a range of values in both teachers and student populations. Vaccination coverage in children in European countries at different dates is reported in **Figure S9**.

We considered values for vaccine effectiveness against infection from the Omicron variant from available estimates for children [77] and adults [78] (**Table S4**). We assumed vaccine effectiveness against transmission to be 20% in both children and adults.

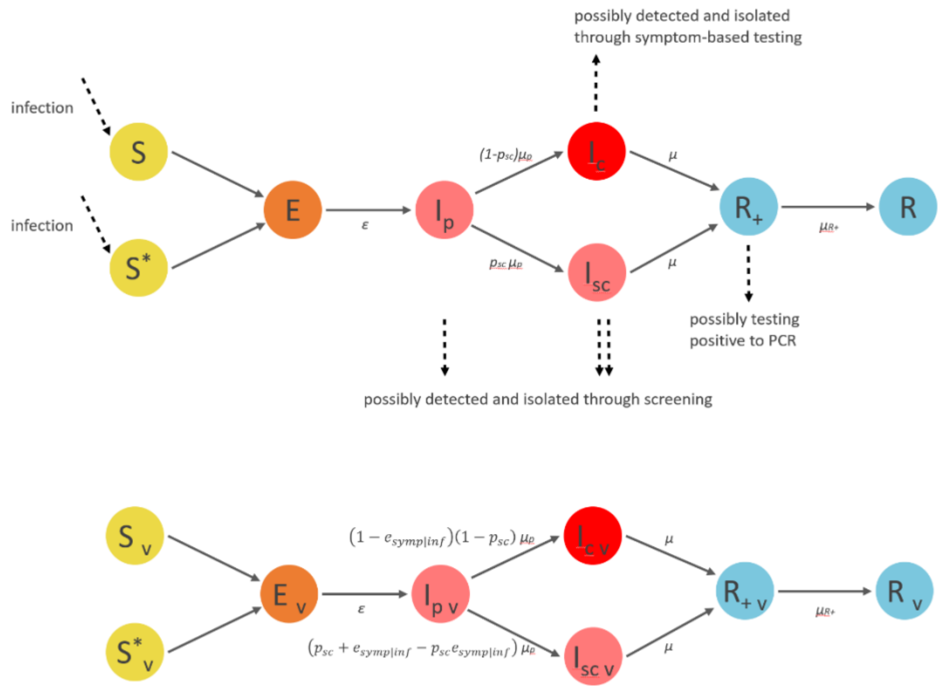

Figure S8. Compartmental scheme with vaccination.

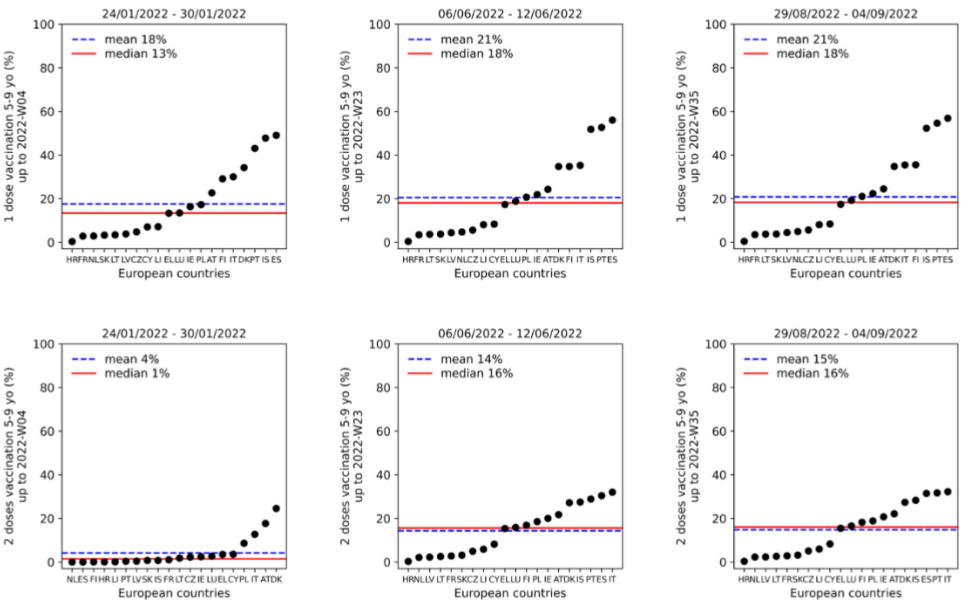

Figure S9. Vaccination coverage of children in Europe. Vaccination coverage refers to one dose (top row), and two doses (bottom row) at three moments in time: January, June and September 2022. Data for Italy from [79] for age class 5-11, data for France is from [80] for age class 5-9, data for other European countries from [81] for age class 5-9. The countries represented are: Austria, Cyprus, Czech Republic, Denmark, France, Greece, Spain, Finland, Croatia, Ireland, Iceland, Italy, Liechtenstein, Lithuania, Luxembourg, Latvia, Netherlands, Poland, Portugal, Slovakia.

Table S4. Vaccine effectiveness (VE) against symptomatic infection estimates considered in the study.

	Vaccine effectiveness against infection	Value	Source
Adults	At 6 months after the second dose	15%	In line with [78]
	Within the first 4 weeks after booster	70%	In line with [78]
Children	Within the first 4 weeks after second dose	50%	In line with [77]
	Lower VE	20%	Assumed to account for a strong waning of immunity

2. Inference framework

To infer weekly introductions w_i at school in France, we used the incidence surveillance data [80] collected during the Omicron peak wave (10/01/2022 – 03/02/2022). We fit the weekly introductions w_i and the starting date t_0 of the simulation. The model fit is performed with a maximum likelihood approach on the observed 7-day rolling incidence rate in the age class 6-10 years old.

The likelihood function is of the form

$$L(Data|\theta) = \prod_{d=d_1}^{d_n} Poiss(\, cases_{obs}(d) \mid cases_{pred}(d))$$

where $\theta = \{w_i,\ t_0\}$ indicates the set of unknown parameters to be estimated, $cases_{obs}(d)$ is the observed total number of positive cases on day d over a 7-day time window, $cases_{pred}(d)$ is the model-predicted total number of positive cases on day d over a 7-day time window, $Poiss(\cdot \mid cases_{pred}(d))$ is the probability mass function of a Poisson distribution with mean $cases_{pred}(d)$, and $[d_1, d_n]$ is the time window considered for the fit.

3. Supplementary results

3.1. Incidence and number of tests per student over time under different introduction conditions

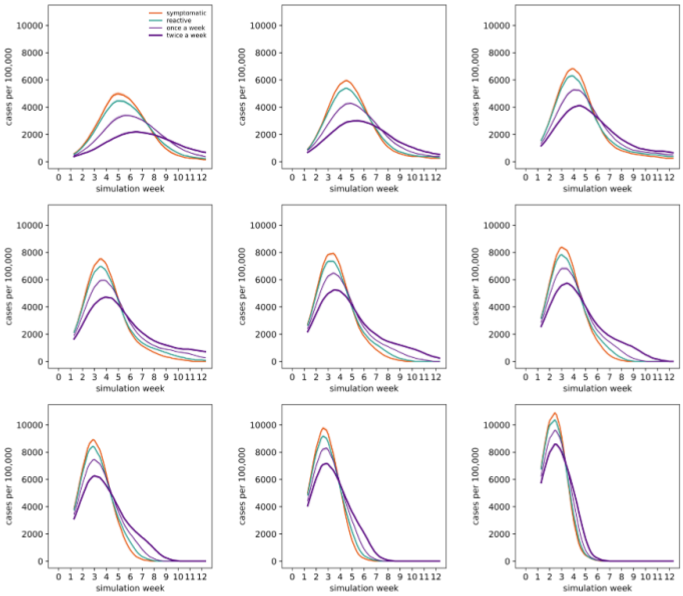

Figure S10. Incidence over time under different school protocols, varying initial incidence levels. Simulated weekly incidence is expressed in number of cases in students per 100,000 over time for increasing values of introduction conditions.

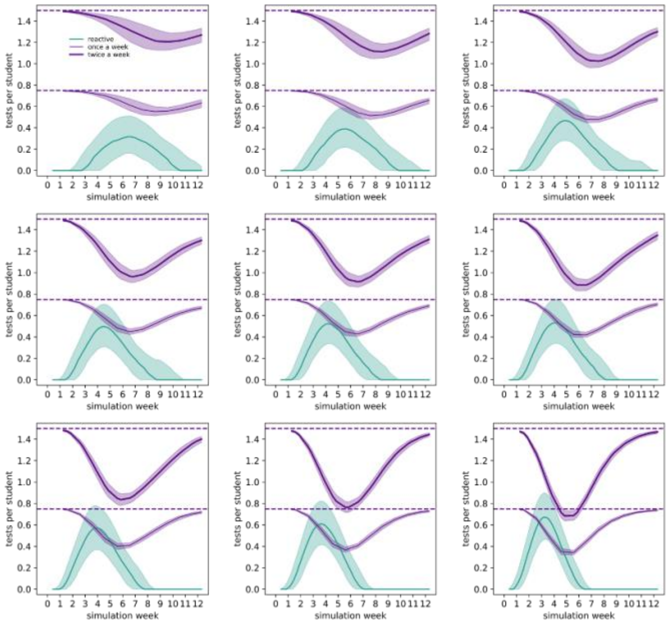

Figure S11. Number of tests per student over time under different school protocols, varying initial incidence levels. Average number of tests per student over time for reactive and regular protocols under the epidemic conditions illustrated in **Figure S10**. The horizontal dashed lines indicate the theoretical values of the demands in number of tests per student in the once and twice per week screening (i.e. imposed by 75% adherence and by frequency). Results are obtained considering the use of nasal LFD tests in both reactive and regular screenings. Shaded areas around the curves correspond to IQR ranges.

3.2. Test needs and schooldays lost vs. percentage of case reduction at varying incidence rates.

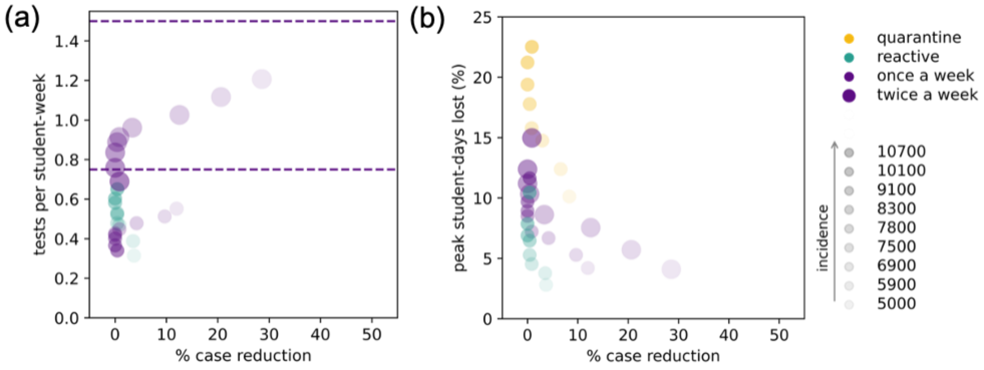

Figure S12. Test needs and schooldays lost vs. percentage case reduction at varying incidence rates. (a) Demand in the number of tests per student-week at peak as a function of the percentage of case reduction achieved by each protocol compared to symptomatic testing (i.e. in absence of interventions). The horizontal dashed lines indicate the theoretical values of the number of tests per student in the once and twice per week screening (i.e. imposed by 75% adherence and the frequency). Dots reduce their transparency for increasing incidence. (b) Percentage of student-days lost as a function of the percentage of case reduction achieved by each protocol. The percentage of case reduction is computed with respect to symptomatic testing. The reactive quarantine of the class is shown as an additional protocol. In panel (b) incidence values in the legend refer to peak incidence of symptomatic testing (i.e. in absence of interventions); the corresponding values for each protocol are plotted in **Figure 2A**.

3.3. Vaccination coverage for children

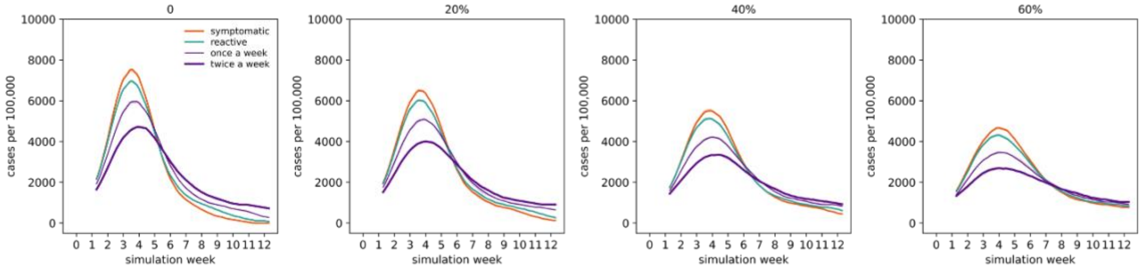

Figure S13. Incidence over time under different school protocols and vaccination coverages in children. Simulated weekly incidence expressed in number of cases in students per 100,000 over time for different protocols, vaccination coverage in children varies between 0 and 60%.

4. Sensitivity analysis

4.1. Sensitivity analysis on vaccination coverage for teachers

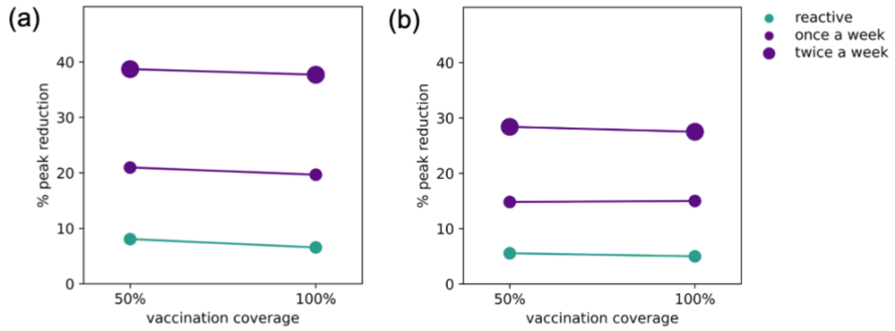

Figure S14. Impact of vaccination coverage with booster for teachers. Percentage of peak reduction achieved by each protocol compared to symptomatic testing (i.e. in absence of interventions) as a function of vaccination coverage in teachers for lower (a) and higher (b) introduction conditions. In panels (a) and (b), peak incidence values in the absence of interventions are 7,500 and 10,100 cases per 100,000 respectively.

4.2. Sensitivity analysis on advantage in transmission rate of Omicron relative to Delta

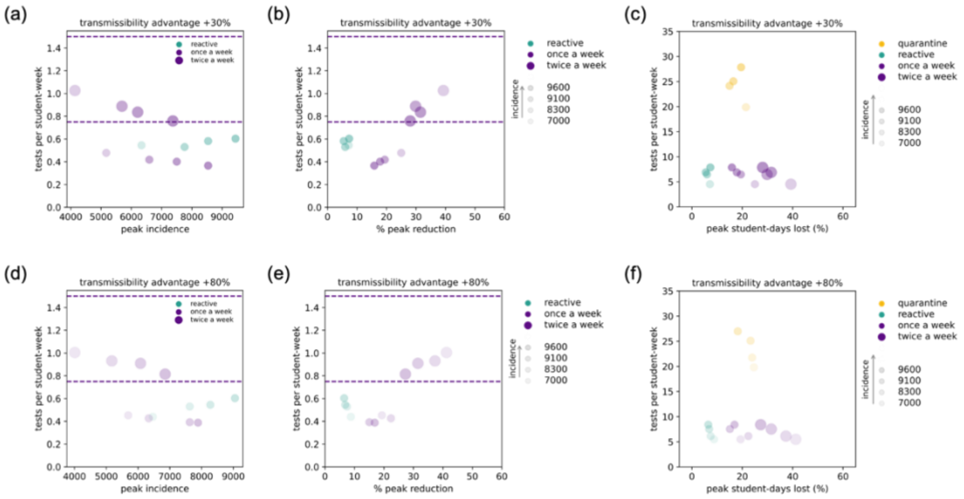

Figure S15. Impact of advantage in transmission rate of Omicron relative to Delta. (a,d) Demand in the number of tests per student-week at peak as a function of the peak incidence (cases in students per 100,000) for the reactive, once per week, and twice per week protocols. The horizontal dashed lines indicate the theoretical values of the number of tests per student in the once and twice per week screening (i.e. imposed by 75% adherence and the frequency). Dots reduce their transparency for increasing incidence. (b,e) Demand in the number of tests per student-week at peak as a function of the percentage of peak reduction achieved by each protocol compared to symptomatic testing (i.e. in absence of interventions). The horizontal dashed lines are as in panels (a,d). Dots transparency code is the same as in panels (a,d). (c,f) Peak percentage of student-days lost as a function of the percentage of peak reduction achieved by each protocol; both quantities are computed with respect to symptomatic testing. The reactive quarantine of the class is shown as an additional protocol. In panels (b,c,e,f) incidence values in the legend refer to peak incidence of symptomatic testing (i.e. in absence of interventions); the corresponding values for each protocol are plotted in panels (a,d). In all panels, peak incidence values in the absence of interventions range between 7,000 and 9,600 cases per 100,000.

4.3. Sensitivity analysis on latent period of Omicron

We considered a latent period of the Omicron with respect to the Delta variant 0.5 days shorter (subsection 1.1). We explored for sensitivity a latent period of the Omicron with respect to the Delta variant of the same length and 1 day shorter.

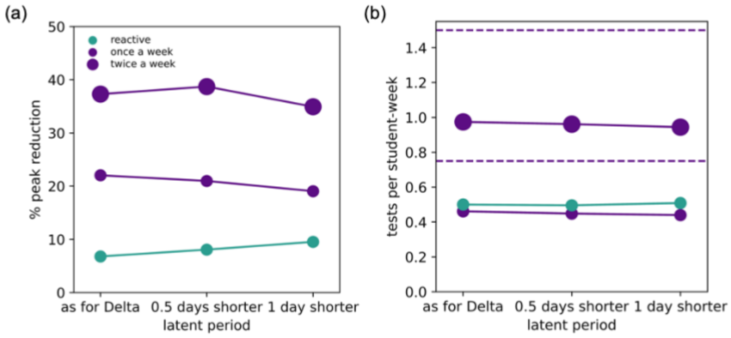

Figure S16. Impact of latent period of Omicron. (a) Percentage of peak reduction achieved by each protocol compared to symptomatic testing (i.e. in absence of interventions) as a function of the latent period when latent period is either as for Delta or 1 day shorter. (b) Demand in the number of tests per student-week at peak as a function of the latent period. The horizontal dashed lines indicate the theoretical values of the number of tests per student in the once and twice per week screening (i.e. imposed by 75% adherence and the frequency). In all panels, peak incidence value in the absence of interventions ranges from 7,400 to 7,800 cases per 100,000.

4.4. Sensitivity analysis on detection probability

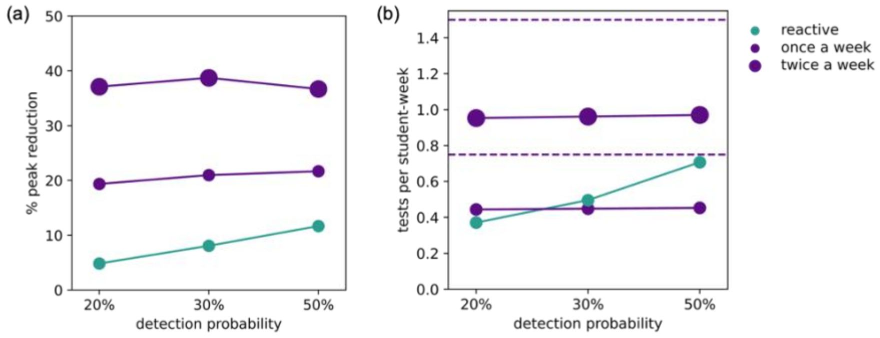

Figure S17. Impact of detection probability. (a) Percentage of peak reduction achieved by each protocol compared to symptomatic testing (i.e. in absence of interventions) as a function of detection probability. (b)

Demand in the number of tests per student-week at peak as a function of the detection probability. The horizontal dashed lines indicate the theoretical values of the number of tests per student in the once and twice per week screening (i.e. imposed by 75% adherence and the frequency). Peak incidence values in the absence of interventions range from 5,100 to 12,300 cases per 100,000.

4.5. Sensitivity analysis on control screening

We considered the reactive protocol applied in France in January 2022, requesting an LFD test at days D0, D2, and D4 to the class of the detected case, following case identification. We explored for sensitivity an LFD test at days D0, D3, and D7 and an LFD test at days D0, D4.

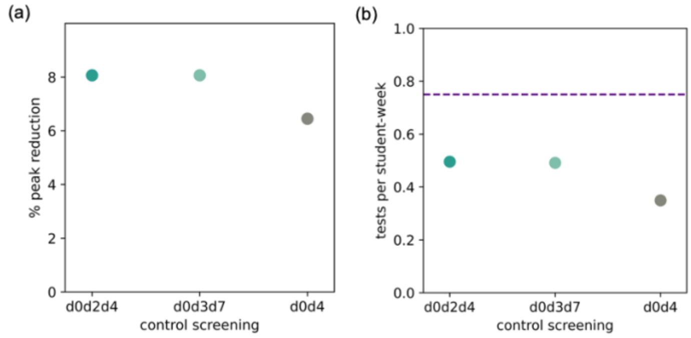

Figure S18. Impact of control screening. (a) Percentage of peak reduction achieved by each protocol compared to symptomatic testing (i.e. in absence of interventions) as a function of number and delays of control screenings. (b) Demand in the number of tests per student-week at peak as a function of number and delays of control screenings. The horizontal dashed lines indicate the theoretical values of the number of tests per student in the once per week screening (i.e. imposed by 75% adherence and the frequency). Peak incidence value in the absence of interventions is around 7,500 cases per 100,000.

5. References

[1] Li R, Pei S, Chen B, Song Y, Zhang T, Yang W, et al. Substantial undocumented infection facilitates the rapid dissemination of novel coronavirus (SARS-CoV-2). Science 2020;368:489–93. https://doi.org/10.1126/science.abb3221.

[2] Qiu X, Nergiz AI, Maraolo AE, Bogoch II, Low N, Cevik M. The role of asymptomatic and pre-symptomatic infection in SARS-CoV-2 transmission—a living systematic review. Clinical Microbiology and Infection 2021;27:511–9. https://doi.org/10.1016/j.cmi.2021.01.011.

[3] Buitrago-Garcia D, Egli-Gany D, Counotte MJ, Hossmann S, Imeri H, Ipekci AM, et al. Occurrence and transmission potential of asymptomatic and presymptomatic SARS-CoV-2 infections: A living systematic review and meta-analysis. PLOS Medicine 2020;17:e1003346. https://doi.org/10.1371/journal.pmed.1003346.

[4] Luo L, Liu D, Liao X, Wu X, Jing Q, Zheng J, et al. Contact Settings and Risk for Transmission in 3410 Close Contacts of Patients With COVID-19 in Guangzhou, China. Annals of Internal Medicine 2020;173:879–87. https://doi.org/10.7326/M20-2671.

[5] Noh JY, Yoon JG, Seong H, Choi WS, Sohn JW, Cheong HJ, et al. Asymptomatic infection and atypical manifestations of COVID-19: Comparison of viral shedding duration. Journal of Infection 2020;81:816–46. https://doi.org/10.1016/j.jinf.2020.05.035.

[6] Ferguson N, Ghani A, Cori A, Hogan A, Hinsley W, Volz E. Report 49: Growth, population distribution and immune escape of Omicron in England. Imperial College London; 2021. https://doi.org/10.25561/93038.

[7] Tindale LC, Stockdale JE, Coombe M, Garlock ES, Lau WYV, Saraswat M, et al. Evidence for transmission of COVID-19 prior to symptom onset. ELife 2020;9:e57149. https://doi.org/10.7554/eLife.57149.

[8] He X, Lau EHY, Wu P, Deng X, Wang J, Hao X, et al. Temporal dynamics in viral shedding and transmissibility of COVID-19. Nature Medicine 2020;26:672–5. https://doi.org/10.1038/s41591-020-0869-5.

[9] Li Q, Guan X, Wu P, Wang X, Zhou L, Tong Y, et al. Early Transmission Dynamics in Wuhan, China, of Novel Coronavirus–Infected Pneumonia. New England Journal of Medicine 2020;382:1199–207. https://doi.org/10.1056/NEJMoa2001316.

[10] Lauer SA, Grantz KH, Bi Q, Jones FK, Zheng Q, Meredith HR, et al. The Incubation Period of Coronavirus Disease 2019 (COVID-19) From Publicly Reported Confirmed Cases: Estimation and Application. Annals of Internal Medicine 2020;172:577–82. https://doi.org/10.7326/M20-0504.
[11] Guan W, Ni Z, Hu Y, Liang W, Ou C, He J, et al. Clinical Characteristics of Coronavirus Disease 2019 in China. New England Journal of Medicine 2020;382:1708–20. https://doi.org/10.1056/NEJMoa2002032.
[12] Backer JA, Klinkenberg D, Wallinga J. Incubation period of 2019 novel coronavirus (2019-nCoV) infections among travellers from Wuhan, China, 20–28 January 2020. Eurosurveillance 2020;25:2000062. https://doi.org/10.2807/1560-7917.ES.2020.25.5.2000062.
[13] Wölfel R, Corman VM, Guggemos W, Seilmaier M, Zange S, Müller MA, et al. Virological assessment of hospitalized patients with COVID-2019. Nature 2020;581:465–9. https://doi.org/10.1038/s41586-020-2196-x.
[14] Ganyani T, Kremer C, Chen D, Torneri A, Faes C, Wallinga J, et al. Estimating the generation interval for coronavirus disease (COVID-19) based on symptom onset data, March 2020. Eurosurveillance 2020;25:2000257. https://doi.org/10.2807/1560-7917.ES.2020.25.17.2000257.
[15] Young BE, Ong SWX, Kalimuddin S, Low JG, Tan SY, Loh J, et al. Epidemiologic Features and Clinical Course of Patients Infected With SARS-CoV-2 in Singapore. JAMA 2020;323:1488–94. https://doi.org/10.1001/jama.2020.3204.
[16] McGee RS, Homburger JR, Williams HE, Bergstrom CT, Zhou AY. Model-driven mitigation measures for reopening schools during the COVID-19 pandemic. PNAS 2021;118. https://doi.org/10.1073/pnas.2108909118.
[17] Kang M, Xin H, Yuan J, Ali ST, Liang Z, Zhang J, et al. Transmission dynamics and epidemiological characteristics of SARS-CoV-2 Delta variant infections in Guangdong, China, May to June 2021. Eurosurveillance 2022;27:2100815. https://doi.org/10.2807/1560-7917.ES.2022.27.10.2100815.
[18] Faes C, Willem L, Franco N, Hens N, Beutels P, Abrams S. SARS-CoV-2 variants and vaccination in Belgium. SIMID consortium; 2022.
[19] Jansen L, Tegomoh B, Lange K, Showalter K, Figliomeni J, Abdalhamid B, et al. Investigation of a SARS-CoV-2 B.1.1.529 (Omicron) Variant Cluster — Nebraska, November–December 2021. MMWR Morb Mortal Wkly Rep 2021;70. https://doi.org/10.15585/mmwr.mm705152e3.
[20] Viner RM, Mytton OT, Bonell C, Melendez-Torres GJ, Ward J, Hudson L, et al. Susceptibility to SARS-CoV-2 Infection Among Children and Adolescents Compared With Adults: A Systematic Review and Meta-analysis. JAMA Pediatrics 2021;175:143. https://doi.org/10.1001/jamapediatrics.2020.4573.
[21] Fontanet A, Tondeur L, Grant R, Temmam S, Madec Y, Bigot T, et al. SARS-CoV-2 infection in schools in a northern French city: a retrospective serological cohort study in an area of high transmission, France, January to April 2020. Eurosurveillance 2021;26:2001695. https://doi.org/10.2807/1560-7917.ES.2021.26.15.2001695.
[22] Thompson HA, Mousa A, Dighe A, Fu H, Arnedo-Pena A, Barrett P, et al. Severe Acute Respiratory Syndrome Coronavirus 2 (SARS-CoV-2) Setting-specific Transmission Rates: A Systematic Review and Meta-analysis. Clinical Infectious Diseases 2021. https://doi.org/10.1093/cid/ciab100.
[23] Davies NG, Klepac P, Liu Y, Prem K, Jit M, Eggo RM. Age-dependent effects in the transmission and control of COVID-19 epidemics. Nature Medicine 2020;26:1205–11. https://doi.org/10.1038/s41591-020-0962-9.
[24] Riccardo F, Ajelli M, Andrianou XD, Bella A, Manso MD, Fabiani M, et al. Epidemiological characteristics of COVID-19 cases and estimates of the reproductive numbers 1 month into the epidemic, Italy, 28 January to 31 March 2020. Eurosurveillance 2020;25:2000790. https://doi.org/10.2807/1560-7917.ES.2020.25.49.2000790.
[25] Shekerdemian LS, Mahmood NR, Wolfe KK, Riggs BJ, Ross CE, McKiernan CA, et al. Characteristics and Outcomes of Children With Coronavirus Disease 2019 (COVID-19) Infection Admitted to US and Canadian Pediatric Intensive Care Units. JAMA Pediatr 2020;174:868. https://doi.org/10.1001/jamapediatrics.2020.1948.
[26] Dong Y, Mo X, Hu Y, Qi X, Jiang F, Jiang Z, et al. Epidemiology of COVID-19 Among Children in China. Pediatrics 2020;145. https://doi.org/10.1542/peds.2020-0702.
[27] Colosi E, Bassignana G, Contreras DA, Poirier C, Boëlle P-Y, Cauchemez S, et al. Screening and vaccination against COVID-19 to minimise school closure: a modelling study. The Lancet Infectious Diseases 2022;22:977–89. https://doi.org/10.1016/S1473-3099(22)00138-4.
[28] Dattner I, Goldberg Y, Katriel G, Yaari R, Gal N, Miron Y, et al. The role of children in the spread of COVID-19: Using household data from Bnei Brak, Israel, to estimate the relative susceptibility and infectivity of children. PLOS Computational Biology 2021;17. https://doi.org/10.1371/journal.pcbi.1008559.

[29] Santé publique France. Séroprévalence du SARS-CoV-2 en population générale, France entière : résultats préliminaires de la semaine 42 de 2021 (18-23 octobre). Santé publique France; 2021.
[30] Stehlé J, Voirin N, Barrat A, Cattuto C, Isella L, Pinton J-F, et al. High-Resolution Measurements of Face-to-Face Contact Patterns in a Primary School. PLOS ONE 2011;6:e23176. https://doi.org/10.1371/journal.pone.0023176.
[31] Vestergaard CL, Génois M. Temporal Gillespie Algorithm: Fast Simulation of Contagion Processes on Time-Varying Networks. PLOS Computational Biology 2015;11:e1004579. https://doi.org/10.1371/journal.pcbi.1004579.
[32] Di Domenico L, Valdano E, Colosi E, Colizza V. Risk of COVID-19 introductions in schools in France week 37 (Sept 7-13, 2020). 2020.
[33] Fox SJ, Lachmann M, Meyers LA. Risks of COVID-19 Introductions as Schools Reopen. The University of Texas at Austin, COVID-19 Modeling Consortium 2020.
[34] Andriamanga C, Minaya-Flores P, Tessier D, Carbonneil C, Zeghari-Squalli N, Dalour S. Méta-analyse de l'intérêt diagnostique des tests RT-PCR salivaires de détection du SARS-CoV-2. Haute Autorité de santé; 2021.
[35] Andriamanga C, Minaya-Flores P, Tessier D, Carbonneil C. Évaluation de l'intérêt diagnostique des tests RT-LAMP réalisés sur système intégré et prélèvement salivaire pour détecter les sujets infectés par le SARS-CoV-2. Haute Autorité de santé; 2021.
[36] Azzi L, Carcano G, Gianfagna F, Grossi P, Gasperina DD, Genoni A, et al. Saliva is a reliable tool to detect SARS-CoV-2. Journal of Infection 2020;81:e45–50. https://doi.org/10.1016/j.jinf.2020.04.005.
[37] Bastos ML, Perlman-Arrow S, Menzies D, Campbell JR. The Sensitivity and Costs of Testing for SARS-CoV-2 Infection With Saliva Versus Nasopharyngeal Swabs. Annals of Internal Medicine 2021;174:501–10. https://doi.org/10.7326/M20-6569.
[38] Butler-Laporte G, Lawandi A, Schiller I, Yao M, Dendukuri N, McDonald EG, et al. Comparison of Saliva and Nasopharyngeal Swab Nucleic Acid Amplification Testing for Detection of SARS-CoV-2: A Systematic Review and Meta-analysis. JAMA Internal Medicine 2021;181:353–60. https://doi.org/10.1001/jamainternmed.2020.8876.
[39] Czumbel LM, Kiss S, Farkas N, Mandel I, Hegyi A, Nagy Á, et al. Saliva as a Candidate for COVID-19 Diagnostic Testing: A Meta-Analysis. Frontiers in Medicine 2020;7:465. https://doi.org/10.3389/fmed.2020.00465.
[40] De Santi C, Jacob B, Kroich P, Doyle S, Ward R, Li B, et al. Concordance between PCR-based extraction-free saliva and nasopharyngeal swabs for SARS-CoV-2 testing [version 2; peer review: 2 approved]. HRB Open Research 2021;4. https://doi.org/10.12688/hrbopenres.13353.1.
[41] Han H, Luo Q, Mo F, Long L, Zheng W. SARS-CoV-2 RNA more readily detected in induced sputum than in throat swabs of convalescent COVID-19 patients. The Lancet Infectious Diseases 2020;20. https://doi.org/10.1016/S1473-3099(20)30174-2.
[42] Hanson KE, Barker AP, Hillyard DR, Gilmore N, Barrett JW, Orlandi RR, et al. Self-Collected Anterior Nasal and Saliva Specimens versus Health Care Worker-Collected Nasopharyngeal Swabs for the Molecular Detection of SARS-CoV-2. Journal of Clinical Microbiology 2020;58:e01824-20. https://doi.org/10.1128/JCM.01824-20.
[43] Iwasaki S, Fujisawa S, Nakakubo S, Kamada K, Yamashita Y, Fukumoto T, et al. Comparison of SARS-CoV-2 detection in nasopharyngeal swab and saliva. Journal of Infection 2020;81:e145–7. https://doi.org/10.1016/j.jinf.2020.05.071.
[44] Kojima N, Turner F, Slepnev V, Bacelar A, Deming L, Kodeboyina S, et al. Self-Collected Oral Fluid and Nasal Swabs Demonstrate Comparable Sensitivity to Clinician Collected Nasopharyngeal Swabs for Coronavirus Disease 2019 Detection. Clinical Infectious Diseases 2020. https://doi.org/10.1093/cid/ciaa1589.
[45] Peeters E, Singh SKDA, Vandesompele J, Mestdagh P, Hutse V, Arbyn M. Rapid systematic review of the sensitivity of SARS-CoV-2 molecular testing on saliva compared to nasopharyngeal swabs. MedRxiv Preprint 2020:2020.08.05.20168716. https://doi.org/10.1101/2020.08.05.20168716.
[46] Sakanashi D, Asai N, Nakamura A, Miyazaki N, Kawamoto Y, Ohno T, et al. Comparative evaluation of nasopharyngeal swab and saliva specimens for the molecular detection of SARS-CoV-2 RNA in Japanese patients with COVID-19. Journal of Infection and Chemotherapy 2021;27:126–9. https://doi.org/10.1016/j.jiac.2020.09.027.
[47] Sasaki T, Inoue O, Ogihara S, Kubokawa K, Oishi S, Shirai T, et al. Detection of SARS-CoV-2 RNA using RT-qPCR in nasopharyngeal swab, saliva, lingual, and buccal mucosal swab. Japanese Journal of Infectious Diseases 2021:JJID.2021.091. https://doi.org/10.7883/yoken.JJID.2021.091.

[48] To KK-W, Tsang OT-Y, Yip CC-Y, Chan K-H, Wu T-C, Chan JM-C, et al. Consistent Detection of 2019 Novel Coronavirus in Saliva. Clinical Infectious Diseases 2020;71:841–3. https://doi.org/10.1093/cid/ciaa149.
[49] Tu Y-P, Jennings R, Hart B, Cangelosi GA, Wood RC, Wehber K, et al. Swabs Collected by Patients or Health Care Workers for SARS-CoV-2 Testing. New England Journal of Medicine 2020. https://doi.org/10.1056/NEJMc2016321.
[50] Vogels CBF, Watkins AE, Harden CA, Brackney DE, Shafer J, Wang J, et al. SalivaDirect: A simplified and flexible platform to enhance SARS-CoV-2 testing capacity. Med 2021;2:263-280.e6. https://doi.org/10.1016/j.medj.2020.12.010.
[51] Wyllie AL, Fournier J, Casanovas-Massana A, Campbell M, Tokuyama M, Vijayakumar P, et al. Saliva or Nasopharyngeal Swab Specimens for Detection of SARS-CoV-2. New England Journal of Medicine 2020. https://doi.org/10.1056/NEJMc2016359.
[52] Yokota I, Shane PY, Okada K, Unoki Y, Yang Y, Inao T, et al. Mass screening of asymptomatic persons for SARS-CoV-2 using saliva. Clinical Infectious Diseases 2020. https://doi.org/10.1093/cid/ciaa1388.
[53] Fronza F, Groff N, Martinelli A, Passerini BZ, Rensi N, Cortelletti I, et al. A Community Study of SARS-CoV-2 Detection by RT-PCR in Saliva: A Reliable and Effective Method. Viruses 2022;14:313. https://doi.org/10.3390/v14020313.
[54] AbdulRahman A, AlBastaki A, AlAwadhi A, Alwazaan A, AlQahtani M. Diagnostic and monitoring utilities of saliva for SARS-CoV-2. MedRxiv Preprint 2020:2020.12.07.20244681. https://doi.org/10.1101/2020.12.07.20244681.
[55] Wells CR, Townsend JP, Pandey A, Moghadas SM, Krieger G, Singer B, et al. Optimal COVID-19 quarantine and testing strategies. Nature Communications 2021;12:356. https://doi.org/10.1038/s41467-020-20742-8.
[56] Miller TE, Beltran WFG, Bard AZ, Gogakos T, Anahtar MN, Astudillo MG, et al. Clinical sensitivity and interpretation of PCR and serological COVID-19 diagnostics for patients presenting to the hospital. The FASEB Journal 2020;34:13877–84. https://doi.org/10.1096/fj.202001700RR.
[57] Smith RL, Gibson LL, Martinez PP, Ke R, Mirza A, Conte M, et al. Longitudinal Assessment of Diagnostic Test Performance Over the Course of Acute SARS-CoV-2 Infection. The Journal of Infectious Diseases 2021;224:976–82. https://doi.org/10.1093/infdis/jiab337.
[58] Bruce EA, Huang M-L, Perchetti GA, Tighe S, Laaguiby P, Hoffman JJ, et al. Direct RT-qPCR detection of SARS-CoV-2 RNA from patient nasopharyngeal swabs without an RNA extraction step. PLOS Biology 2020;18:e3000896. https://doi.org/10.1371/journal.pbio.3000896.
[59] Ford L, Whaley MJ, Shah MM, Salvatore PP, Segaloff HE, Delaney A, et al. Antigen Test Performance Among Children and Adults at a SARS-CoV-2 Community Testing Site. Journal of the Pediatric Infectious Diseases Society 2021;10:1052–61. https://doi.org/10.1093/jpids/piab081.
[60] Pollock NR, Jacobs JR, Tran K, Cranston AE, Smith S, O'Kane CY, et al. Performance and Implementation Evaluation of the Abbott BinaxNOW Rapid Antigen Test in a High-Throughput Drive-Through Community Testing Site in Massachusetts. Journal of Clinical Microbiology 2021;59:e00083-21. https://doi.org/10.1128/JCM.00083-21.
[61] Pollock NR, Tran K, Jacobs JR, Cranston AE, Smith S, O'Kane CY, et al. Performance and Operational Evaluation of the Access Bio CareStart Rapid Antigen Test in a High-Throughput Drive-Through Community Testing Site in Massachusetts. Open Forum Infectious Diseases 2021;8. https://doi.org/10.1093/ofid/ofab243.
[62] Prince-Guerra JL. Evaluation of Abbott BinaxNOW Rapid Antigen Test for SARS-CoV-2 Infection at Two Community-Based Testing Sites — Pima County, Arizona, November 3–17, 2020. MMWR Morb Mortal Wkly Rep 2021;70. https://doi.org/10.15585/mmwr.mm7003e3.
[63] Shah MM, Salvatore PP, Ford L, Kamitani E, Whaley MJ, Mitchell K, et al. Performance of Repeat BinaxNOW Severe Acute Respiratory Syndrome Coronavirus 2 Antigen Testing in a Community Setting, Wisconsin, November 2020–December 2020. Clinical Infectious Diseases 2021;73:S54–7. https://doi.org/10.1093/cid/ciab309.
[64] Pilarowski G, Marquez C, Rubio L, Peng J, Martinez J, Black D, et al. Field Performance and Public Health Response Using the BinaxNOWTM Rapid Severe Acute Respiratory Syndrome Coronavirus 2 (SARS-CoV-2) Antigen Detection Assay During Community-Based Testing. Clinical Infectious Diseases 2020. https://doi.org/10.1093/cid/ciaa1890.
[65] Young S, Taylor SN, Cammarata CL, Varnado KG, Roger-Dalbert C, Montano A, et al. Clinical Evaluation of BD Veritor SARS-CoV-2 Point-of-Care Test Performance Compared to PCR-Based Testing and versus the Sofia 2 SARS Antigen Point-of-Care Test. Journal of Clinical Microbiology 2020;59:e02338-20. https://doi.org/10.1128/JCM.02338-20.

[66] Nikolai O, Rohardt C, Tobian F, Junge A, Corman VM, Jones TC, et al. Anterior nasal versus nasal mid-turbinate sampling for a SARS-CoV-2 antigen-detecting rapid test: does localisation or professional collection matter? Infectious Diseases 2021;53:947–52. https://doi.org/10.1080/23744235.2021.1969426.
[67] Zhou Y, O'Leary TJ. Relative sensitivity of anterior nares and nasopharyngeal swabs for initial detection of SARS-CoV-2 in ambulatory patients: Rapid review and meta-analysis. PLOS ONE 2021;16:e0254559. https://doi.org/10.1371/journal.pone.0254559.
[68] Basu A, Zinger T, Inglima K, Woo K, Atie O, Yurasits L, et al. Performance of Abbott ID Now COVID-19 Rapid Nucleic Acid Amplification Test Using Nasopharyngeal Swabs Transported in Viral Transport Media and Dry Nasal Swabs in a New York City Academic Institution. Journal of Clinical Microbiology 2020;58:e01136-20. https://doi.org/10.1128/JCM.01136-20.
[69] Berenger BM, Fonseca K, Schneider AR, Hu J, Zelyas N. Sensitivity of Nasopharyngeal, Nasal and Throat Swab for the Detection of SARS-CoV-2. MedRxiv Preprint 2020:2020.05.05.20084889. https://doi.org/10.1101/2020.05.05.20084889.
[70] LeBlanc JJ, Heinstein C, MacDonald J, Pettipas J, Hatchette TF, Patriquin G. A combined oropharyngeal/nares swab is a suitable alternative to nasopharyngeal swabs for the detection of SARS-CoV-2. Journal of Clinical Virology 2020;128:104442. https://doi.org/10.1016/j.jcv.2020.104442.
[71] Lee RA, Herigon JC, Benedetti A, Pollock NR, Denkinger CM. Performance of Saliva, Oropharyngeal Swabs, and Nasal Swabs for SARS-CoV-2 Molecular Detection: a Systematic Review and Meta-analysis. Journal of Clinical Microbiology 2021;59:e02881-20. https://doi.org/10.1128/JCM.02881-20.
[72] Péré H, Podglajen I, Wack M, Flamarion E, Mirault T, Goudot G, et al. Nasal Swab Sampling for SARS-CoV-2: a Convenient Alternative in Times of Nasopharyngeal Swab Shortage. Journal of Clinical Microbiology 2020;58:e00721-20. https://doi.org/10.1128/JCM.00721-20.
[73] Han MS, Choi EH, Chang SH, Jin B-L, Lee EJ, Kim BN, et al. Clinical Characteristics and Viral RNA Detection in Children With Coronavirus Disease 2019 in the Republic of Korea. JAMA Pediatrics 2021;175:73. https://doi.org/10.1001/jamapediatrics.2020.3988.
[74] Smith LE, Potts HWW, Amlôt R, Fear NT, Michie S, Rubin GJ. Adherence to the test, trace, and isolate system in the UK: results from 37 nationally representative surveys. BMJ 2021;372:n608. https://doi.org/10.1136/bmj.n608.
[75] Centers for Disease Control and Prevention. Interim Guidance for Antigen Testing for SARS-CoV-2. Centers for Disease Control and Prevention 2020. https://www.cdc.gov/coronavirus/2019-ncov/lab/resources/antigen-tests-guidelines.html (accessed April 12, 2021).
[76] MIUR. Nota tecnica - Indicazioni per l'individuazione e la gestione dei contatti di casi di infezione da SARS-CoV-2 in ambito scolastico. Ministero dell'Istruzione Ministero dell'Università e della Ricerca 2021. https://www.miur.gov.it/documents/20182/0/Nota+tecnica+-+Indicazioni+per+l%E2%80%99individuazione+e+la+gestione+dei+contatti+di+casi+di+infezione+da+SARS-CoV-2+in+ambito+scolastico.pdf/838ef7ff-225c-2cbe-f3b8-a90448e10fc9?version=1.0&t=1636204411355 (accessed February 1, 2022).
[77] Cocchio S, Zabeo F, Tremolada G, Facchin G, Venturato G, Marcon T, et al. COVID-19 Vaccine Effectiveness against Omicron Variant among Underage Subjects: The Veneto Region's Experience. Vaccines 2022;10:1362. https://doi.org/10.3390/vaccines10081362.
[78] Kirsebom FCM, Andrews N, Stowe J, Toffa S, Sachdeva R, Gallagher E, et al. COVID-19 vaccine effectiveness against the omicron (BA.2) variant in England. The Lancet Infectious Diseases 2022;22:931–3. https://doi.org/10.1016/S1473-3099(22)00309-7.
[79] Commissario straordinario per l'emergenza Covid-19 - Presidenza del Consiglio dei Ministri. Covid-19 Opendata Vaccini 2022. https://github.com/italia/covid19-opendata-vaccini (accessed February 3, 2022).
[80] Géodes - Santé publique France - Indicateurs : cartes, données et graphiques n.d. https://geodes.santepubliquefrance.fr/#view=map2&c=indicator (accessed April 12, 2021).
[81] ECDC. European Centre for Disease Prevention and Control - Data on COVID-19 vaccination in the EU/EEA. European Centre for Disease Prevention and Control 2021. https://www.ecdc.europa.eu/en/publications-data/data-covid-19-vaccination-eu-eea (accessed November 10, 2021).
